# Thromboaspiration of a left-sided bioprosthetic valve thrombosis by a mini-access: the Lausanne novel procedure

**DOI:** 10.3389/fcvm.2024.1371692

**Published:** 2024-07-04

**Authors:** Ziyad Gunga, Vladimir Rubimbura, Denise Oberson, Pierre Monney, Xavier Bechtold, Zied Ltaief, Valentina Rancati, Eric Eeckhout, Matthias Kirsch

**Affiliations:** ^1^ Cardiac Surgery Department, Lausanne University Hospital (CHUV), Lausanne, Switzerland; ^2^ Cardiology Department, Lausanne University Hospital (CHUV), Lausanne, Switzerland; ^3^ Cardiovascular Perfusionist, Lausanne University Hospital (CHUV), Lausanne, Switzerland; ^4^ Anesthesiology Department, Lausanne University Hospital (CHUV), Lausanne, Switzerland

**Keywords:** valve thrombosis, bioprosthesis, cardiac surgery, complications, thrombus, thromboaspiration, Occlutech, Lausanne procedure

## Abstract

Left-sided bioprosthesis valve thrombosis (LSBVT) is a challenging complication necessitating invasive interventions. In this study, we introduce a novel, minimally invasive approach. We used a cerebral embolic protection system and an Occlutech cannula connected to an extracorporeal circuit, providing safer thrombus aspiration compared to the AngioVac system. This technique offers a promising alternative for high-risk patients with LSBVT.

## Learning objectives

Understand the clinical challenges associated with left-sided bioprosthesis valve thrombosis (LSBVT), including its multifactorial causes and the limitations of traditional treatment options.
Introduce the Lausanne procedure, a pioneering minimally invasive approach for treating LSBVT, including its surgical technique and use of cerebral embolic protection systems ensuring neurological security.
Recognize the safety considerations and technical features of the procedure, including the use of the Occlutech® cannula as the aspirating cannula, as an alternative to the AngioVac-system.
Examine the potential advantages of the Lausanne procedure, such as reduced complications, shorter hospital stays, and improved patient outcomes, in comparison with conventional interventions (redo-surgery).

## Introduction

Left-sided bioprosthetic valve thrombosis (LSBVT) is a serious but often overlooked complication that can occur in both surgical and transcatheter prostheses (1). The multifactorial underlying causes include inadequate postoperative antithrombotic therapy, prothrombotic conditions, ventricular dysfunction, and atrial fibrillation. Although oral anticoagulation is the gold-standard treatment, thrombolysis and traditional surgical interventions remain available options. However, these treatments are invasive and can be associated with significant morbidity (2).

**Figure 1:**
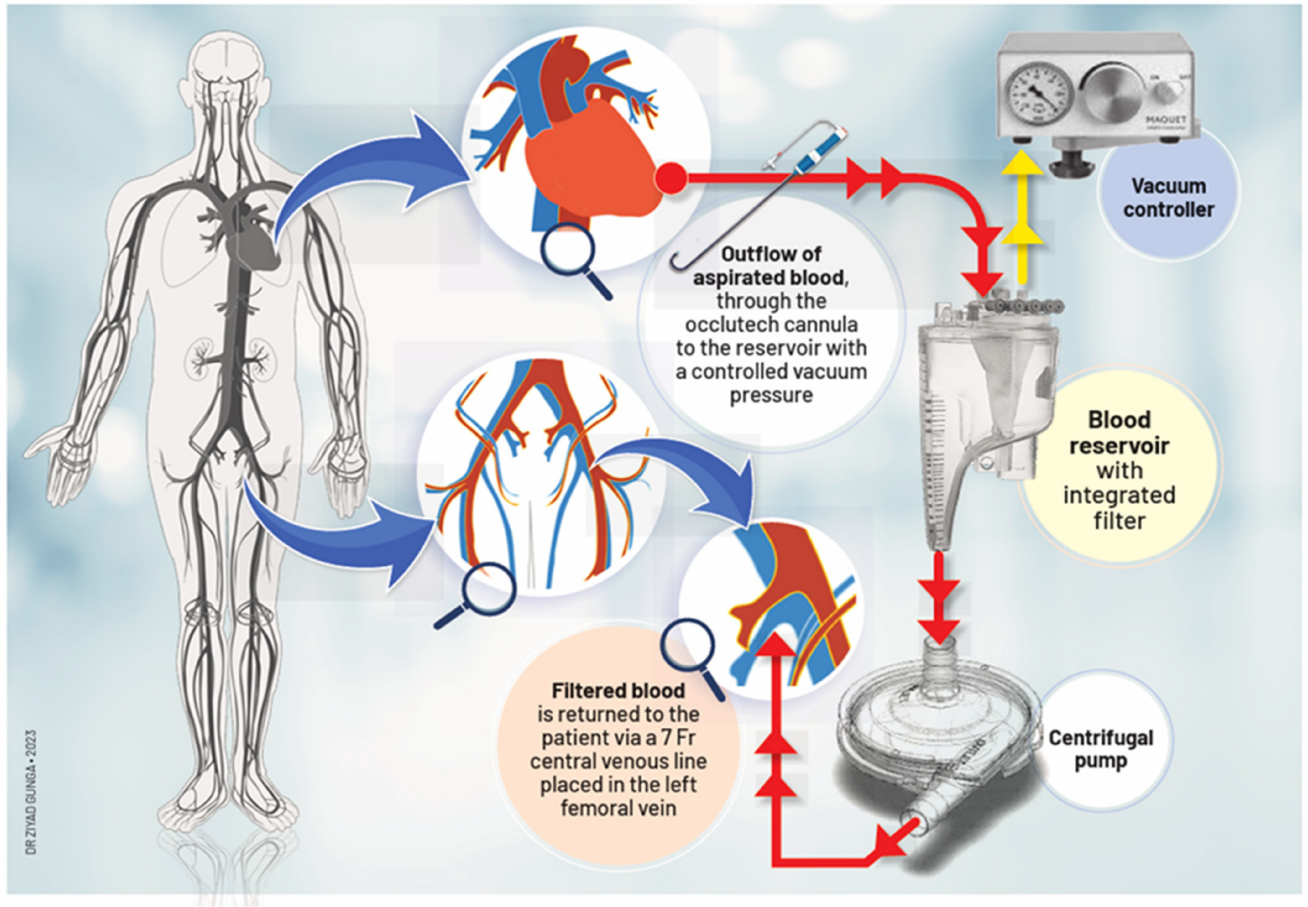
The Lausanne novel thromboaspiration procedure design.

To address this challenge, we propose a novel procedure via a minimally invasive approach to treat LSBVT (Figure 1). Our method may provide a safer and more effective alternative for high-risk patients.

## Case presentation

**Figure 2:**
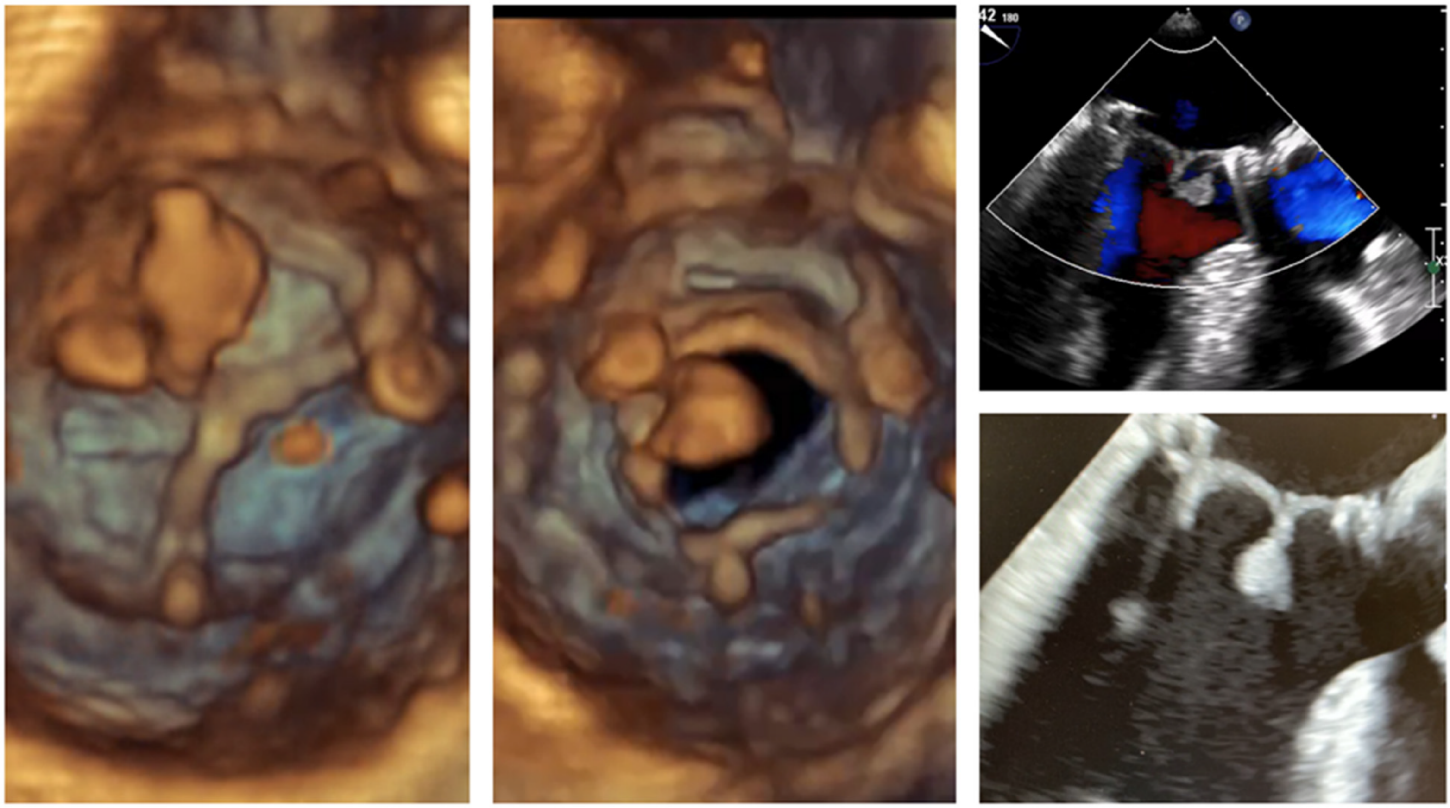
TEE images showing a mobile mass attached to a mitral bioprosthesis.

A 54-year-old male patient with a normal ejection fraction underwent mitral valve replacement with a 33 mm Carpentier-Edwards Magna-Ease pericardial bioprosthesis in September 2022. At the 2-month postoperative echocardiographic follow-up, a motile mass was incidentally detected, pedunculated to the bioprosthesis, and crisscrossing the valve with each heartbeat (Video 1, Figure 2). Notably, the patient remained asymptomatic throughout this period, and the bioprosthesis did not exhibit any restricted leaflet motion, nor was there occlusion of the left ventricular outflow tract (LVOT). Despite the absence of clinical or laboratory evidence for endocarditis, a broad-spectrum empiric antibiotherapy was initiated for a period of 3 weeks conjointly with therapeutic-dose anticoagulation using heparin. The echocardiographic control showed no alteration in size and aspect of the mass. The patient adamantly refused to undergo a redo on-pump cardiac surgery. A thrombolytic therapy was discarded because of the unpredictable risk of embolization (3). However, because of its bulky nature (18 mm × 11 mm), hypermobility, resistance to anticoagulation, and the neurological threat it posed, we were urged to devise a way to remove the thrombus via minimally invasive access, as per the patient's desire (Figure 1).

## Surgical technique

**Figure 3:**
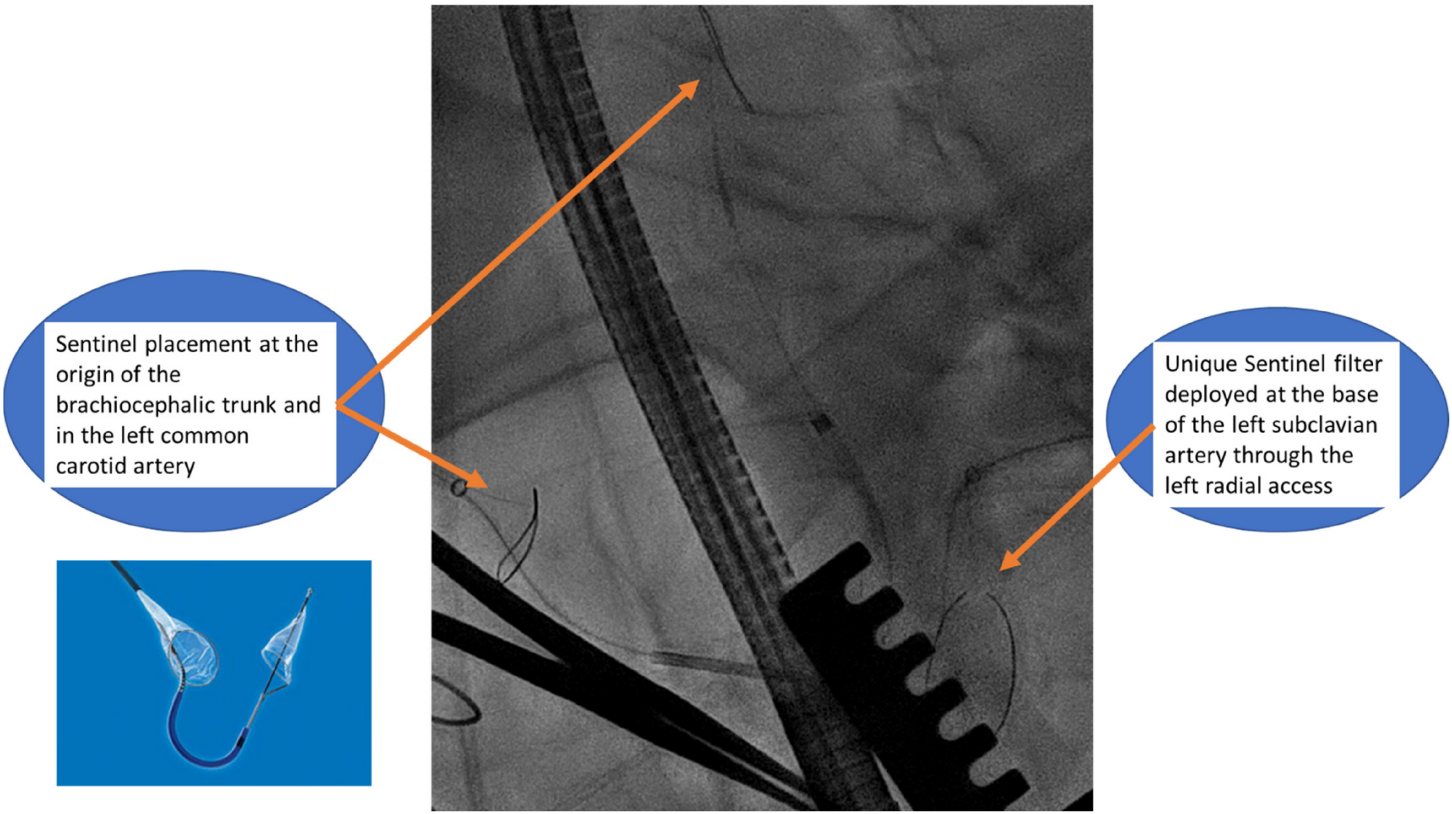
Fluoroscopic image highlighting the placement of Sentinel cerebral protective system.

The patient was placed supine with a roller pad positioned vertically under the left scapula to provide a slight rightward tilt. The patient was intubated with a double-lumen tube, and the left radial artery and the right and left groins were prepared in the sterile surgical field. A 4 cm transverse incision was made 1 cm below the areolar line, directed toward the apex, which was identified using echocardiographic guidance. The fifth intercostal space was opened, and the absence of lung adhesion was confirmed after selective intubation. A soft tissue retractor and a rib spreader retractor were placed. The pericardium was opened over 4 cm facing the apex. There was not much adhesion. Stay sutures were placed allowing optimal exposure of the apex. The center of the apex was pinpointed by transesophageal echocardiography (TEE). Two concentric purse-string sutures were placed using Ethibond-3/0 and buttressed over polytetrafluoroethylene (PTFE)-felt pledgets, with adequate bites taken through the muscle but refrained from penetrating the left ventricular cavity. Systemic heparin was then administered to the patient before the placement of the Sentinel cerebral protection system (SCPS), which is a dual filter designed to protect against embolization. Under fluoroscopic guidance, the device was inserted through the right radial access with the proximal filter deployed first at the origin of the brachiocephalic trunk. The distal segment of the catheter was then articulated to navigate through the aortic arch and into the left common carotid artery for deployment of the distal filter. Another filter is placed similarly at the base of the subclavian artery through the left radial access to protect the left vertebral artery (Figure 3).

**Figure 4:**
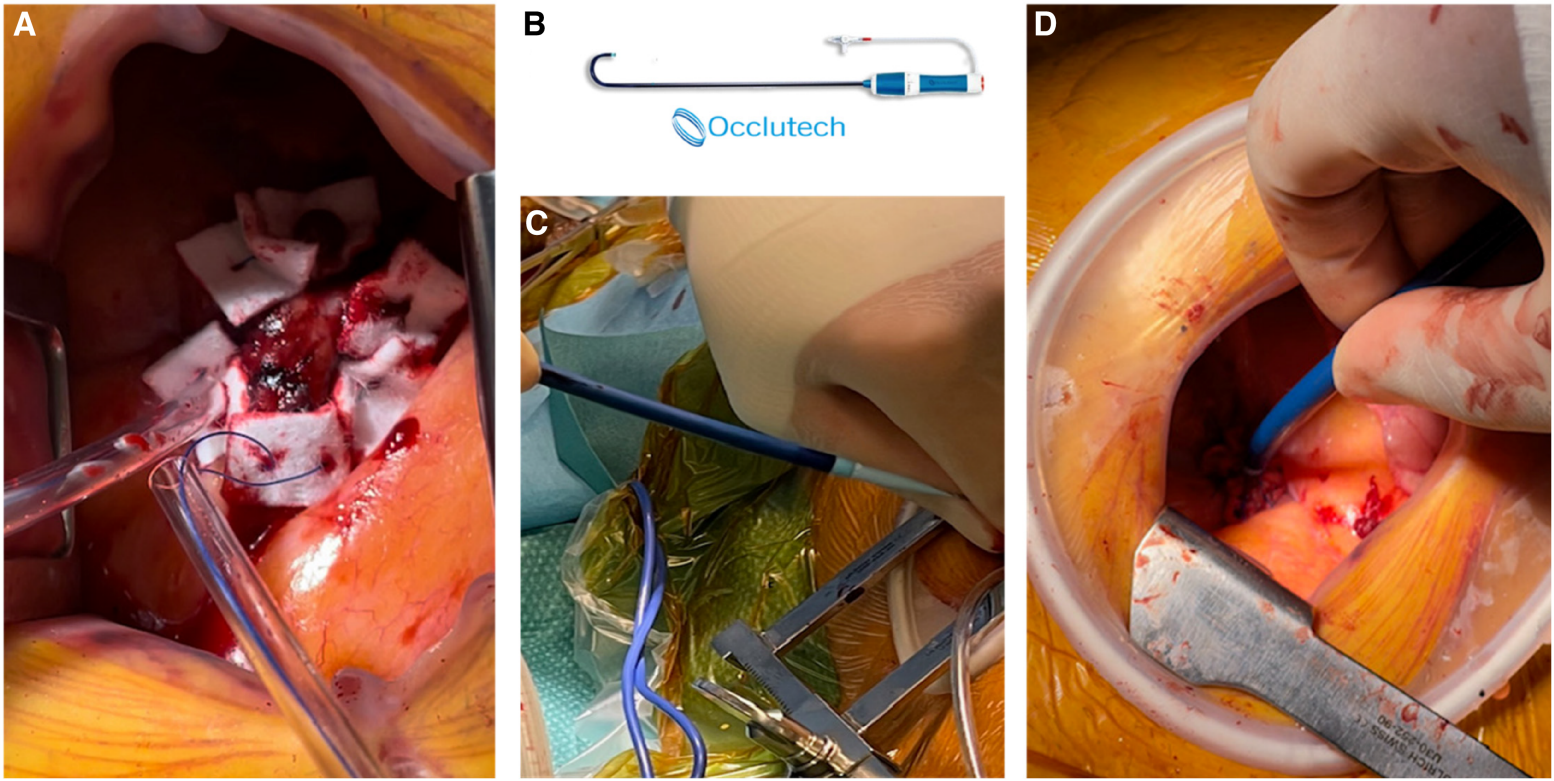
(**A**) Two concentric purse-string sutures on the apex. (**B**) Occlutech cannula. (**C**,**D**) Apical insertion of the cannula with its dilatator.

**Figure 5:**
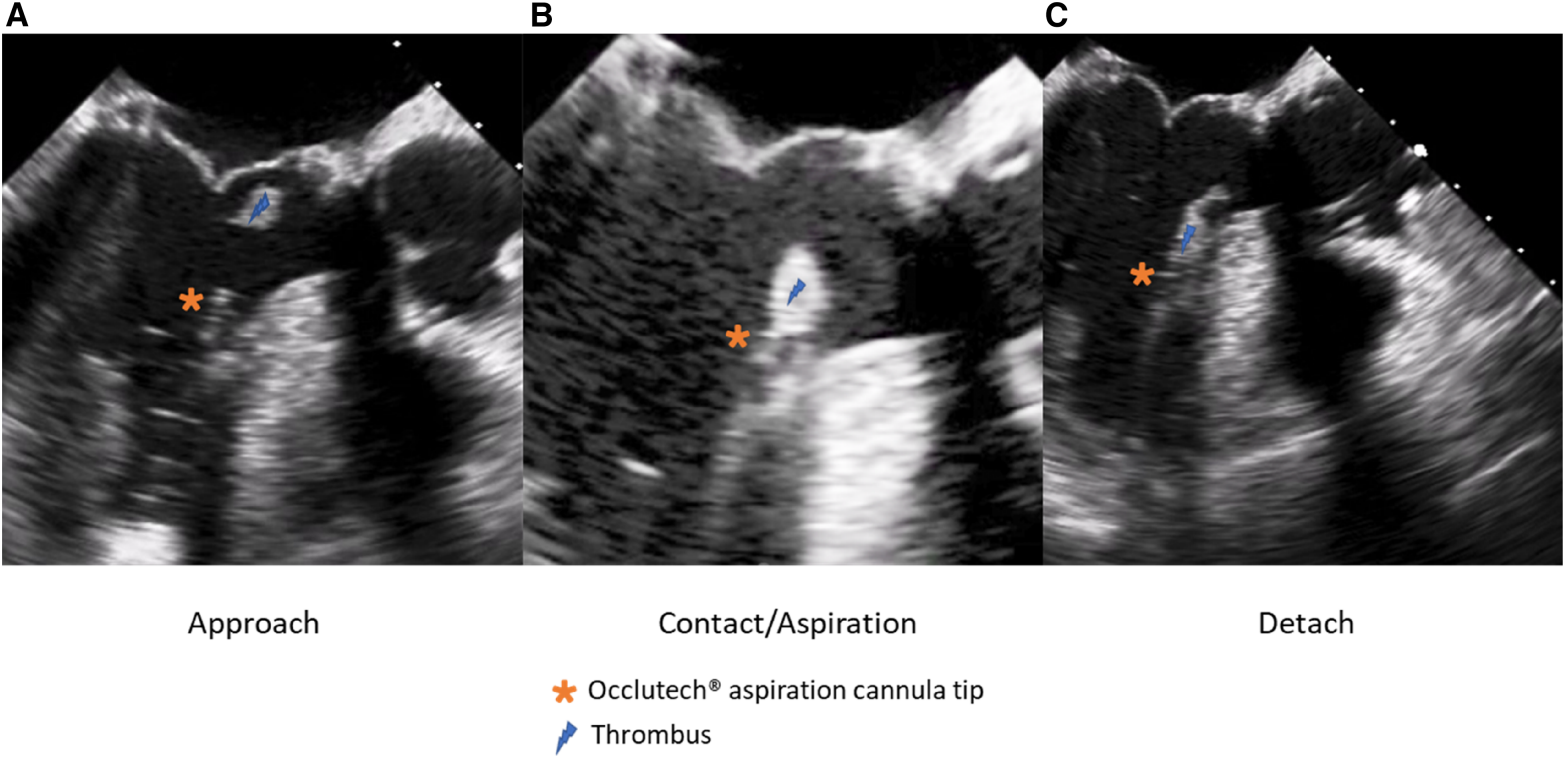
Echo-guided description of the thromboaspiration procedure in three steps (**A–C**).

The patient's hemodynamic stability was closely monitored throughout the procedure. TEE and fluoroscopic control were used to guide the following steps. The apex was punctured with a needle, and a soft guidewire was inserted antegrade across the aortic valve making sure to avoid the thrombus. A 14 Fr Occlutech steerable guiding sheath (Occlutech International AB, Helsingborg, Sweden) was inserted with its dilatator (Figure 4), which was subsequently removed once in place in the LVOT. The proximal part of the Occlutech was connected to a pediatric extracorporeal circuit including a reservoir with a filter, a centrifugal pump, and a reinjection route via a 7 Fr catheter placed in the left femoral vein to return the aspirated blood. The Occlutech adjustable tip was meticulously moved forward into the LVOT abutting the thrombus. The extracorporeal machine was started and the thrombus was aspirated (Video 2, Figure 5) into the Occlutech aperture with 300 ml of blood, which was filtered and restored back to the patient. After confirming the absence of any residual thrombus and assessing good valvular function, the Occlutech sheath was removed, and the apical sutures were gently tied for hemostasis, avoiding excessive tension that could cause myocardial tearing. Biological glue VeraSeal Fibrin Sealant (Johnson & Johnson MedTech), as a suture support, was sprinkled over the apex, and the pericardium was closed without any tension. A local analgesic was injected into the intercostal space to alleviate postoperative pain. A chest tube was placed in the left thoracic cavity. The ribs were gently approached after verifying the re-expansion of the left lung, and the wound was closed in a regular fashion. The filter in the reservoir presented sedimented microthrombi, resulting from the aspiration of the thrombus. The surgery was uneventful, with immediate extubation and successful removal of the mass confirmed via echocardiography. The chest tube drain was removed safely on the following day, and the patient was discharged home. The patient had a smooth postoperative course and remained free of relapse during the first-year follow-ups, indicating a successful outcome.

## Discussion

Prosthetic valve thrombosis (PVT) is a critical complication following valvular replacement, associated with high morbidity and mortality. The occurrence of obstructive PVT in mechanical valves ranges between 0.3% and 1.3% per patient-year, with thromboembolic events, such as systemic emboli, happening at a rate of 0.7%–6% per patient-year. Non-obstructive PVT is quite common postoperatively, with TEE studies showing incidences up to 10% (4). Mechanical valves are more prone to PVT than biological ones, with a higher incidence in right-sided valves compared to left-sided valves and in mitral valves compared to aortic valves.

The predisposition to thrombus formation involves a complex interplay of hemodynamic, surface, and hemostatic factors. Hemodynamic factors include low cardiac output, prosthesis malpositioning, anatomical position of the prosthesis, the prosthetic hemodynamic profile, and hyperviscosity. Surface factors include incomplete endothelialization of the prosthesis, leaflet damage or deterioration, stent fractures, and malpositioning of the prosthesis. Hemostatic factors encompass both primary and secondary hypercoagulable states. Primary states include conditions such as factor V Leiden, prothrombin gene mutation, and deficiencies in antithrombin, protein C, or protein S. Secondary states include atrial fibrillation, malignancy, disseminated intravascular coagulation, antiphospholipid syndrome, cardiomyopathy, nephrotic syndrome, high estrogen levels, sickle cell anemia, and smoking. Additionally, suboptimal anticoagulation, particularly in the early postoperative period (less than 3 months) in patients with mechanical prostheses or surgical bioprostheses, significantly elevates the risk of thrombus formation (5).

The conventional treatment for PVT often involves surgery, with outcomes influenced by symptom severity, clinical status, logistical factors, and surgeon experience. Urgent surgery has an overall mortality rate of 10%–15%, which can increase to 25% in certain cases. Surgical options include valvular replacement or thrombectomy. Onorati et al. (6) reported significant risks associated with redo mitral surgery, including a 12.5% operative mortality rate and complications such as acute myocardial infarction (5.9%), stroke (4.9%), acute respiratory insufficiency (14.8%), pneumonia (7.0%), acute renal insufficiency (16.1%), reintervention for bleeding (7.6%), massive transfusion (28.0%), and the need for a permanent pacemaker (10.1%). For small, recent thrombi (less than 1 cm or 0.8 cm^2^) with mild symptoms [New York Heart Association (NYHA) Classes I–II], a trial of intravenous unfractionated heparin for 24–48 h is often attempted. Fibrinolysis, introduced in the 1970s, shows 82% efficacy but carries a 10% mortality rate and a 12.5% rate of systemic emboli and major bleeding. Contraindications to fibrinolysis, furthermore, include a history of intracranial bleeding, recent cranial trauma, recent gastrointestinal or genitourinary bleeding, hemorrhagic retinopathy, large mobile thrombi, severe hypertension, and recent major surgery. A meta-analysis found higher mortality in surgical treatment compared to thrombolysis (18.1% vs. 6.6%), although the analysis was based on observational studies. Post-thrombolysis, the risk of mobile prosthetic valve thrombosis (MPVT) recurrence remains a concern, with the HATTUSHA study reporting a 2.4% recurrence risk due to incomplete thrombus lysis and the pannus-thrombus association (7).

Clearly, there is insufficient evidence to guide the management of LSBPVT (2). Recent observational studies suggest that most cases, whether obstructive or non-obstructive, can be effectively treated with anticoagulant, obviating the need for surgery or fibrinolysis (2, 3). However, for patients who experience anticoagulation-resistant thrombosis, have excessive surgical risks, or who express concerns about conventional cardiac surgery (or redo), our innovative technique may offer a secure and efficacious alternative.

The safe execution of our procedure relies firstly on the use of cerebral embolic protection devices to mitigate the risk of cerebral embolization (8). When manipulating guidewires within the left chambers, there is a possibility of dislodging the thrombus, leading to potential neurological risks. However, by safeguarding the innominate and left common carotid arteries with the Sentinel system and placing a second Sentinel in the left subclavicular artery to protect the left vertebral artery, we successfully avert any potential neurological threats. Another alternative might be the use of a TriGuard neuroprotection device (TriGuard HDH, Keystone Heart, Tampa, FL, USA), which is positioned in the aortic arch, providing full coverage of the supra-aortic vessels altogether, deployed from an 8 Fr sheath via a femoral access. We preferred the Sentinel to TriGuard for the latter only deflects debris while the Sentinel catches them (9). When considering which device to use, the choice often depends on the specific clinical scenario, physician preference, and the patient's individual needs. It's important to weigh the pros and cons of each device based on the specific procedure and patient history (Table 1).

**Table 1:** Outlines the pros and cons of the two main cerebral embolic protection devices.

Aspect	Filter sentinel	TriGuard
Pros	- Proven track record in various clinical settings	- Provides coverage to all three main cerebral vessels (right and left carotid, vertebral)
	- Relatively easy to deploy in practice	- Offers protection to a wider area of the brain
	- Can effectively capture embolic debris	- Reduces the risk of cerebral ischemia during procedures
	- May be compatible with various catheter types and sizes	- Advanced design may improve protection and efficiency
Cons	- May not cover all cerebral vessels, leaving some risk of embolic events	- Slightly more complex design may require additional training for proper use
	- Potential for incomplete coverage or incomplete debris capture	- Cost may be higher than other options
	- Limited data on its long-term safety and effectiveness in some scenarios	- Still relatively new compared to other options, so long-term data may be more limited

In a few reported cases, the AngioVac system (AngioDynamics, Latham, NY, USA) has been utilized off-label to treat left-sided thrombosis, due to its demonstrated efficacy in removing intravascular and intracardiac thrombus, primarily from the right-sided cavities (10–12). However, given the safety concerns surrounding this system, we have developed a novel approach that employs the Occlutech steerable guiding sheath as the aspirating cannula. This device is connected to an extracorporeal pediatric circuit, without the need for large arterial bore access, since the filtered blood is safely returned via a 7 Fr catheter through the femoral vein. Our novel approach is believed to be a safer alternative to the AngioVac system, which has been criticized for its association with device-related complications, including trauma, bleeding, decreased hemoglobin levels, and distal embolization. Insertion of the 22 Fr cannula through a 26 Fr introducer sheath necessitates large bore access and may cause bleeding (13). Additionally, initiating the AngioVac system requires hemodynamic stabilization by increasing preload, which can dilute the blood and decrease hemoglobin levels. Moreover, the rigidity of the second-generation 22 Fr AngioVac cannula can make it difficult to navigate safely. In contrast, our approach utilizes the Occlutech sheath, which is equipped with bidirectional deflection technology, allowing for up to 180° of deflection without kinking. This feature enables more precise positioning in space and enhances safety.

Dandu et al. (13) conducted a thorough investigation into the complications and failure causes associated with the AngioVac system through post-marketing surveillance utilizing the Manufacturer and User Facility Device Experience (MAUDE) database. Their analysis revealed that among the reported adverse events, pulmonary embolism (PE) emerged as the most prevalent clinical complication, accounting for 34 cases (36.6%). Additionally, perforation was reported in 16 cases (17.2%), arrhythmia in 4 cases (4.3%), stroke in 3 cases (3.2%), and hematoma in 1 case (1.1%). These findings underscore the imperative of thoroughly assessing the safety profile of the AngioVac system and its potential ramifications on patient outcomes when considering various thromboaspiration options.

Most reported cases involving the AngioVac system were conducted with concurrent venoarterial extracorporeal membrane oxygenation (ECMO) as mechanical circulatory support (14). However, this approach exposes patients to the various lethal complications of ECMO, including circuit cavitation, air embolism, thrombus, limb ischemia, vessel perforation or dissection, and pseudoaneurysm (15). Our approach avoids the use of ECMO and thus reduces the risk of such complications (Table 2).

**Table 2:** The safety considerations and technical features of the Lausanne novel procedure.

Advantages	Occlutech cannula	Details
Minimally invasive access	Yes	- Its 14 Fr size allows for a less traumatic transapical approach hence preserving the myocardium at the apex - Less risk of bleeding after retrieving
Precision and flexibility	High	- Bidirectional deflection up to 180° without kinking ensures precise positioning and maneuverability
Reduced risk of complications	Yes	- Smaller access points and an extracorporeal circuit minimize bleeding, trauma, and distal embolization risks
No need for concomitant ECMO	Yes	- Avoids the complications associated with ECMO, such as circuit cavitation, air embolism, and vessel perforation
Safe blood return	Yes	- Aspirated blood is safely returned via a pediatric extracorporeal circuit, maintaining hemodynamic stability
Avoidance of AngioVac issues	Yes	- Addresses AngioVac-related complications such as bleeding and difficulty navigating the rigid cannula - Prevention of adverse events: pulmonary embolism, arrhythmia, stroke, hematoma

After achieving a favorable outcome with our procedure, we have identified potential avenues for future approaches to reduce the risks associated with open thrombectomy. Specifically, we are considering utilizing a transapical approach to aspirate floating aortic arch thrombi, which is a high-risk condition. Additionally, we can leverage the Occlutech sheath, originally designed for delivering patent foramen ovale closure system, to execute safely a transeptal approach for thromboaspiration in the left atrium or appendage. By exploring and implementing this cutting-edge technique, we can improve patient outcomes.

## Conclusion

Our preliminary result demonstrates that this minimally invasive approach is highly effective in our case for treating LSBVT, with fewer complications than traditional interventions. This method offers several advantages, including shorter hospital stays, reduced recovery time, and fewer postoperative complications. This new approach may represent a breakthrough in the treatment of LSBVT, offering hope to patients who were previously deemed unsuitable for invasive interventions. Further clinical trials are needed to confirm the efficacy and safety of this procedure, but our initial result is highly promising.

## Data Availability

The original contributions presented in the study are included in the article/Supplementary Material, further inquiries can be directed to the corresponding author.

## References

[1] Lim WY Lloyd G Bhattacharyya S. Mechanical and surgical bioprosthetic valve thrombosis. Heart. (2017) 103(24):1934–41. 10.1136/heartjnl-2017-311856 28780576

[2] Pislaru SV. Bioprosthetic valve thrombosis: not as simple as it looks. JACC Case Rep. (2022) 4(22):1464–6. 10.1016/j.jaccas.2022.09.003 36444175 PMC9700070

[3] Sachdev S Bardia N Nguyen L Omar B. Bioprosthetic valve thrombosis. Cardiol Res. (2018) 9(6):335–42. 10.14740/cr789 30627283 PMC6306127

[4] Roudaut R Serri K Lafitte S. Thrombosis of prosthetic heart valves: diagnosis and therapeutic considerations. Heart. (2007) 93(1):137–42. 10.1136/hrt.2005.071183 17170355 PMC1861363

[5] Dangas GD Weitz JI Giustino G Makkar R Mehran R. Prosthetic heart valve thrombosis. J Am Coll Cardiol. (2016) 68(24):2670–89. 10.1016/j.jacc.2016.09.958 27978952

[6] Onorati F Mariscalco G Reichart D Perrotti A Gatti G De Feo M Hospital outcome and risk indices of mortality after redo-mitral valve surgery in potential candidates for transcatheter procedures: results from a European Registry. J Cardiothorac Vasc Anesth. (2018) 32(2):646–53. 10.1053/j.jvca.2017.09.039 29325846

[7] Özkan M Gündüz S Güner A Kalçık M Gürsoy MO Uygur B Thrombolysis or surgery in patients with obstructive mechanical valve thrombosis: the multicenter HATTUSHA study. J Am Coll Cardiol. (2022) 79(10):977–89. 10.1016/j.jacc.2021.12.027 35272803

[8] Haussig S Linke A Mangner N. Cerebral protection devices during transcatheter interventions: indications, benefits, and limitations. Curr Cardiol Rep. (2020) 22(9):96. 10.1007/s11886-020-01335-9 32651654 PMC7351861

[9] Testa L Latib A Casenghi M Gorla R Colombo A Bedogni F. Cerebral protection during transcatheter aortic valve implantation: an updated systematic review and meta-analysis. J Am Heart Assoc. (2018) 7(10):e008463. 10.1161/JAHA.117.008463 29728369 PMC6015324

[10] Memon S Goldman S Hawthorne KM Gnall EM. Percutaneous transeptal mitral valve endocarditis debulking with AngioVac aspiration system. Catheter Cardiovasc Interv. (2022) 100(4):667–73. 10.1002/ccd.30319 35907255

[11] Shadman S Heyliger S Watts C Aghili N. Successful impella-assisted suction thrombectomy of right heart thrombus via the AngioVac device: advantages, limitations, and alternatives. Catheter Cardiovasc Interv. (2021) 97(6):1296–300. 10.1002/ccd.29545 33576557

[12] Fiocco A Colli A Besola L. Case report: treatment of left-sided valve endocarditis using the transapical AngioVac system and cerebral embolism protection device: a case series. Front Cardiovasc Med. (2023) 10:1121488. 10.3389/fcvm.2023.1121488 37063967 PMC10097912

[13] Dandu C Alhusain R Patel D Naughton R Chalek A Polina A TCT-173 Complications and failure modes of the AngioVac thrombectomy system: insight from MAUDE database. J Am Coll Cardiol. (2022) 80(12_Supplement):B70. 10.1016/j.jacc.2022.08.202

[14] Souka A Sukhija R Dimitri DS Imran Arif I. Angiovac debulking of bioprosthetic valve. J Am Coll Cardiol. (2021) 77(18_Supplement_1):2383. 10.1016/S0735-1097(21)03738-4

[15] Zangrillo A Landoni G Biondi-Zoccai G Greco M Greco T Frati G A meta-analysis of complications and mortality of extracorporeal membrane oxygenation. Crit Care Resusc. (2013) 15(3):172–8. 10.1016/s1441-2772(23)01792-1 23944202

